# Physiological Chemistry

**Published:** 1898-01

**Authors:** John A. Miller

**Affiliations:** Professor of chemistry, Niagara University


### PHYSIOLOGICAL CHEMISTRY.

Reported by JOHN A. MILLER, Ph. D.,
Professor of chemistry, Niagara University.

### DIGESTIBILITY OF CACAO BUTTER AND OF BUTTER FROM COW'S MILK.

BOUROT and F. Jean (*Compt. Rend.*, 1896 ; *J. Chem. Soc.*, 1897). Cacao butter is used as a food in many countries, and the present experiments show that it has a high nutritive value. Ordinary butter was digested to the extent of 95.8 and cacao butter to that of 98 per cent.; in both cases, therefore, a very small residue of fat was found in the feces.

---

### COAGULATING AND TOXIC PROPERTIES OF THE LIVER.

A. Mairet and Vires (*Compt. Rend.*, 1896 ; *J. Chem. Soc.*, 1897). An aqueous extract of rabbit's liver was injected intravenously into other rabbits and found in certain doses to cause death ; the blood was found to be coagulated in the heart and veins. By heating the extract a coagulum is obtained ; this contains the substance which produces the intravascular clotting ; the filtrate is, however, still toxic, although it does not coagulate the blood.

---

### TRANSFORMATION OF FAT INTO CARBOHYDRATE IN THE ORGANISM.

J. B. Auguste Chauveau (*Compt. Rend.*, 1896 ; *J. Chem. Soc.*, 1897). In a starving animal and during hibernation, the sugar which still persists in the blood must originate from either fat or proteid. Further, in hibernation Regnault and Reiset showed that the respiratory quotient was very low and the great excess of oxygen retained over carbon dioxide given out leads to an actual increase in weight of the animal, but it loses its fat.

---

### BIOLOGICAL RELATION OF CHLOROPHYLL AND HEMOGLOBIN.

MARCELLUS NENCKI (*Ber.* 29 ; *J. Chem. Soc.*, 1897). Schunck and Marchlewski have shown that phylloporphyrin, $C_{16} H_{18} N_2 O$, a substance obtained from chlorophyll, is nearly related to hematoporphyrin, $C_{16} H_{18} N_2 O_3$ ; hemin and phyllotaonin are also similar and form similar compounds, this similarity extending to spectroscopic appearances. It appears, therefore, that the basis of blood pigment and leaf pigment is the same. In the view of the Darwinian hypothesis, and the essential unity of living things, such a discovery is of value.

---

### FORMATION OF UREA BY OXIDATION.

F. HOFMEISTER (*Arch. Exp. Path. Pharm.*, 37 ; *J. Chem. Soc.*, 1897). With the exception of Béchamp's unrecognised attempts, the formation of urea from albumin or other nitrogenous substances has not been proved. Schultzen and Nencki showed that the nitrogen of amido-acids is excreted almost entirely as urea, and Knieriem proved that even ammonium salts are changed into urea in the animal organism. The most general view, therefore, of the origin of urea is that the final products of oxidation, carbon dioxide and ammonia form urea with elimination of water. Drechsel has shown that, by hydrolysis, only a small portion of the nitrogen of albumin is eliminated as urea.

By oxidation of a great variety of substances with potassium permanganate the author has obtained urea, often even in considerable quantity. The substance was oxidised in aqueous solution, with addition of ammonia and ammonium sulphate, by means of a quantity of potassium permanganate, nearly sufficient to convert it into carbon dioxide, water and urea. Decolorisation took place in times varying from a quarter of an hour to several days. The liquid was then filtered ; the filtrate evaporated almost to dryness at 40–50°, and the crystalline mass digested 10–12 hours, with 96 per cent. alcohol. The filtrate was evaporated, and to the residue, dissolved in alcohol, half its volume of ether was added and the whole then filtered and evaporated. The presence of urea in the residue was proved by preparation of the nitrate and its microscopical examination, or, when in sufficient quantity, by determination of the melting point and estimation of the nitrogen. Urea was proved to have been formed in the cases of hydrocyanic acid, thiocyanic acid, formamide, glycocine, oxamic acid, aspartic acid, asparagine, leucine, gelatin, egg albumin, methyl alcohol, ethylene glycol, glycollic acid, acetone, lactic acid, malic acid, tartaric acid and pyrogallol.

The substances which, on oxidation with permanganate, did not form urea, such as acetamide and oxamide, are also unchanged in the animal organism ; whereas, oxamic acid which yielded urea is also oxidised to urea in the body without previous formation of oxalate. It is still uncertain, however, as to whether this natural synthesis of urea is one of oxidation and elimination of water, but such an assumption does away with the necessity of attributing a special function to the liver, as this organ is found to be especially capable of oxidising fatty substances. The author's observation that the liver contained no cyanic acid is opposed to the view that the formation of urea is analogous to its production from ammonium cyanate.

---

### ACTION OF SUPRARENAL EXTRACTS.

SWALE VINCENT (*Proc. Physiol. Soc.*, 1897 ; *J. Chem. Soc.*, 1897). Large doses of extracts of the suprarenals of mammals, injected subcutaneously, caused death ; the most marked symptom is muscular paralysis of central origin. Blood in the urine, and from the nose, occasionally convulsions, and fall of temperature, were also observed. Glycerine extracts cause local ulceration. Extracts of the cortex of the gland, or of the liver, spleen and kidney are inactive. After a sub-fatal dose, a partial immunity is set up.

---

### ACTION OF OXALIC ACID AND ITS DERIVATIVES ON THE KIDNEYS.

W. EBSTEIN and A. NICOLAIER (*Virchow's Archiv.*, 1897 ; *J. Chem. Soc.*, 1897). The investigation was undertaken with a view to the artificial production of urinary calculi. This was not accomplished, and the present communication relates to the action of oxalic acid and certain of its derivatives on the kidneys when administered in a succession of small doses to dogs and rabbits. Oxalic acid was found to cause the appearance of a deposit of calcium oxalate in the urinary tubules ; these deposits are frequently visible to the naked eye. Oxamic acid is eliminated as a calcium salt in the urine.

### THE WORK OF DIGESTION AND THE EXCRETION OF NITROGEN IN THE URINE.

N. V. Riazantseff (*Arch. des Sci. bial. St. Petersburg*, 4 ; *J. Chem. Soc.*, 1897). The increase in the urinary nitrogen which immediately follows a meal is believed to be due to the increased work of digestion. Foods which produce an increased activity of the secreting glands act in this way more efficaciously than those which produce less activity, but even acidified water (introduced, in a dog, into the stomach by a fistula,) causes the glands to secrete, and this is followed by a rise in the nitrogen excreted in urine.

### ACETONURIA.

Felix Hirschfeld (*Centr. Klin. Med.*, 17 ; *J. Chem. Soc.*, 1897). In healthy persons a diet of proteid and fat leads to an increase of acetone in the urine. The addition of carbohydrate to the diet causes this to disappear. This is explained by its "sparing" action on proteid metabolism. The same holds in the acetonuria of disease and explains the occurrence of acetone in diabetics, in whom carbohydrate metabolism is upset.

